# Stress myocardial perfusion imaging by cardiac magnetic resonance provides strong prognostic value to cardiac events in patients with diabetes

**DOI:** 10.1186/1532-429X-13-S1-O87

**Published:** 2011-02-02

**Authors:** Otavio R Coelho-Filho, François-Pierre Mongeon, Michael Jerosch-Herold, Raymond Y Kwong

**Affiliations:** 1 Brigham and Women's, Boston, MA, USA

## Background

In patients with diabetes mellitus, coronary artery disease (CAD) is a major cause of mortality and results in substantial morbidity. Non-invasive detection of CAD in diabetic patients is challenged by silent ischemia and microvascular disease. Pharmacological stress CMR perfusion imaging (CMRPI) may identify evidence of flow limiting CAD at high resolution and tissue contrast. We therefore sought to test the hypothesis that stress CMRPI can provide robust prognostication to identify diabetic patients at high risk for major adverse cardiac events (MACE).

## Methods/results

Stress CMRPI was performed on 138 consecutive patients with diabetes (54 females, mean age 61±12.3 years) referred for assessment of ischemia. Rest and vasodilator stress CMRPI were performed each using a bolus of 0.1mmol/Kg of gadolinium, followed by late gadolinium enhancement (LGE) and cine imaging. Myocardial ischemia was defined by the presence of a stress induced perfusion defect, reversible at rest, in the absence of LGE in the same territory. At a median follow up of 21 months (IQR 30 months), 21 MACE (15%) had occurred (14 cardiac deaths and 7 AMIs).

**Figure 1:**
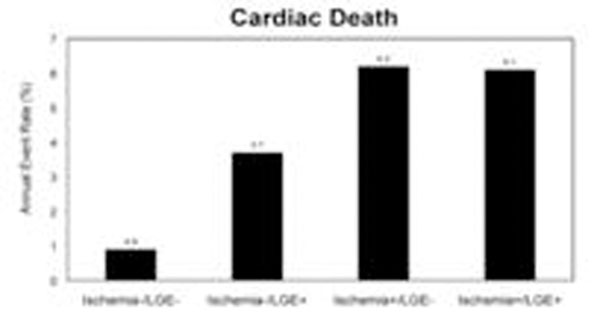


**Figure 2:**
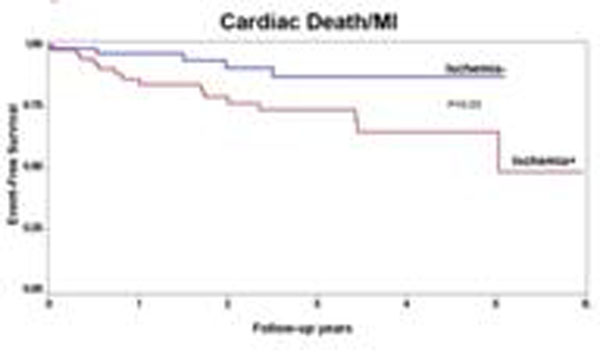


By univariable analysis, the presence of myocardial ischemia by CMRPI portended to 3-fold increase in MACE hazards (HR=2.9, P=0.03). The absence of myocardial ischemia and LGE by CMRPI indicated a low annual rate of cardiac death (0.9%, Figure 1). Patients with myocardial ischemia by CMRPI demonstrated a significantly reduced MACE-free survival (p=0.03, Figure 2). Adjusting for age and presence of LGE, myocardial ischemia by CMRPI maintains a strong adjusted association with MACE (adjusted HR 3.3, P=0,04). The segmental myocardial ischemia score was the only variable selected (HR=1.15, p=0.001, per segment) in the best overall model for MACE by stepwise selection (p<0.01 as criterion for entry and stay).

## Conclusion

Stress CMRPI provides robust prognostication for major cardiac events in patients with diabetes. While a CMR study negative in both myocardial ischemia and LGE indentifies diabetic patients with lower event rates for MACE, the presence of myocardial ischemia by CMRPI indentifies a subgroup of diabetic patients at high risk for cardiac events.

